# Treatment of neuropathic pain in cancer survivors: a scoping review of pharmacological, exercise, and psychosocial interventions

**DOI:** 10.2340/ao.v65.45347

**Published:** 2026-04-24

**Authors:** Ellen Lund Schaldemose, Bolette Skjødt Rafn, Pernille Envold Bidstrup, Agon Olloni, Katrin Schättiger, Peter Christensen, Cæcilie Borregaard Myrhøj, Maja Johannsen Lindberg, Christoffer Johansen, Sandra Jensen, Ida Hovdenak, Sasja Jul Håkonsen, Lise Ventzel

**Affiliations:** a Department of Oncology, Vejle Hospital, University Hospital of Southern Denmark, Vejle, Denmark; b Danish Cancer Society National Research Center for Cancer Survivorship and Treatment Late Effects (CASTLE), Department of Oncology, Copenhagen University Hospital, Rigshospitalet, Copenhagen, Denmark; c Department of Clinical Medicine, University of Copenhagen, Copenhagen, Denmark; d Psychological Aspects of Cancer, Danish Cancer Institute, Denmark; e Department of Oncology, Odense University Hospital, Odense, Denmark; f Department of Internal medicine, University Hospital of Southern Denmark, Sønderborg, Denmark; g Danish Cancer Society National Research Centre on Survivorship and Late Adverse Effects after Cancer in Pelvic Organs, Aarhus, Denmark; h Department of Surgery, Aarhus University Hospital, Aarhus, Denmark; i Department of Clinical Medicine, Aarhus University, Aarhus, Denmark; j Department of Hematology, Copenhagen University Hospital, Rigshospitalet, Copenhagen, Denmark; k Department of Psychology and Behavioral Sciences, Aarhus University, Aarhus, Denmark; l Department of Regional Health Research, Faculty of Health Sciences, University of Southern Denmark, Vejle, Denmark; m The Danish Healthcare Quality Institute (DHQI), Aarhus, Denmark

**Keywords:** Neuropathic pain, exercise, psychological interventions, pharmacological treatment, cancer survivors

## Abstract

**Background and purpose:**

Neuropathic pain is a debilitating late effect among cancer survivors. This scoping review aims to provide an overview of pharmacological, psychological, and exercise interventions for neuropathic pain among cancer survivors and to identify further relevant research areas.

**Patient/material and methods:**

PubMed, PsychInfo, and EMBASE were systematically searched for studies published from January 2004 to January 2026 and abstract and full text screening was carried out. The target population was cancer survivors who had completed primary treatment and have no active disease. Neuropathic pain was defined as a) a mean pain intensity the last week/month of ≥ 3 at a numerical rating scale (0 = no pain, 10 = worst pain), and b) symptoms of neuropathy, or c) neuropathic pain diagnosed by an experienced neurologist.

**Results:**

Of the 956 systematic reviews/guidelines and 604 original studies identified, 11 pharmacological, two psychological and three studies on exercise were eligible. Most of the studies included patients with breast cancer.

Duloxetine was effective in reducing neuropathic pain from painful chemotherapy-induced neuropathy and gabapentin + concomitant morphine compared to morphine alone reduced neuropathic pain in cancer survivors with neuropathic pain due to radiation therapy, and surgery. Mindfulness-based cognitive behavioral therapy showed no effect after correction for multiple comparisons. Exercise interventions were useful in both reducing neuropathic pain as well as neuropathic symptoms.

**Interpretation:**

This scoping review found evidence for pharmacological treatment of neuropathic pain in cancer survivors, could not make any conclusion on psychological treatment, and exercise interventions show promising effects. Further research on interdisciplinary treatment of neuropathic pain among cancer survivors is needed.

## Introduction

With advances in cancer screening, diagnosis, treatment and survival [1, 2], the number of individuals living with physical and psychological late effects to cancer and cancer management is increasing [3].

One of the debilitating late effects of cancer management is chronic pain, including neuropathic pain, which can substantially interfere with daily functioning and overall quality of life [4, 5]. There is no treatment of neuropathic pain (i.e. the neurological damage cannot be reversed). However, several treatment approaches are available to reduce pain symptoms, yet [6, 7], it is still unclear how to most efficiently deal with this late effect.

Neuropathic pain is caused by a lesion or dysfunction in the somatosensory nervous system. This disruption can lead to spontaneous pain signals or exaggerated responses to normally non-painful stimuli, resulting in characteristic symptoms such as burning, shooting, or electric shock-like sensations. To diagnose neuropathic pain, the Neuropathic Pain Special Interest Group (NeuPSIG) of the International Association of the Study of Pain (IASP) have published a diagnosis and grading system from ‘possible’ to ‘definite’ neuropathic pain [8].

Together with neuropathic pain, general symptoms of neuropathy (regardless of etiology) such as reduced sensitivity, reduced balance, prickling or tingling or the feeling on walking on cotton wool also contribute to the neuropathic symptomatology [9].

The etiology of cancer-related neuropathic pain is diverse, including neurological damage due to chemotherapy (chemotherapy-induced peripheral neuropathy [CIPN]) or nerve damage following surgery or radiation therapy [10]. The prevalence of cancer-related neuropathic pain has been described across a wide range of cancer populations. In a systematic review from 2012 encompassing 13,683 patients with various types of cancer, the prevalence of neuropathic pain among patients with ongoing cancer treatment was 19–38% [11]. Among cancer survivors, the estimated prevalence of neuropathic pain has been reported to be between 4 and 60% when caused by surgery [12–16], between 30 and 60% for painful CIPN [11, 17, 18], and between 2 and 25% for post-radiation therapy neuropathic pain [19–21].

Across different etiologies, there is no pharmacological treatment to reduce neuropathic *symptoms*, but only to reduce neuropathic pain [22]. Exercise interventions have, however, been shown to alleviate neuropathic symptoms and pain and improve muscular strength and balance [23], although there is a need of larger and more detailed studies [24]. Less research has studied psychological interventions such as cognitive behavioral therapy (CBT) [25]. However, there are numerous studies conducted in psycho-oncology settings that demonstrate the efficacy of particularly CBT on the pain experience, although not on neuropathic pain specifically [26, 27].

International consensus guidelines include pharmacological recommendations for treatment of neuropathic pain among patients with cancer [11, 12]. Former reviews have mainly focused on pharmacological treatment of neuropathic pain in either cancer patients across the illness trajectory [12], patients with active cancer [13] or cancer survivors with chronic pain (both nociceptive and neuropathic) [14]. No previous reviews have focused exclusively on cancer survivors who have completed cancer treatment and do not have active disease.

Cancer-related neuropathic pain is a complex, multi-dimensional condition that spans sensory, functional, and psychological domains [28–32] and existing interventions have only partly addressed distinct therapeutic domains. We conducted a comprehensive review to systematically map the evidence on pharmacological, psychological, and exercise interventions for neuropathic pain among cancer survivors. As such, our findings can be useful in identifying any existing gaps in knowledge and to prioritize areas of further research in the interdisciplinary treatment of neuropathic pain in cancer survivors.

## Patients/material and methods

The scoping review was based on systematic literature searches performed as part of the development of a Danish national guideline on treatment of neuropathic pain in cancer survivors coordinated by the Danish Multidisciplinary Cancer Group [15]. The scoping review was performed and reported according to the Preferred Reporting Items for Systematic Reviews and Meta-Analyses – scoping reviews (PRISMA-ScR) guidelines [16]. We did not make a PROSPERO registration.

### Eligibility criteria

Search strategies were developed in consultation with a Clinical Guidelines Methodology Specialist (SH) and the research team members. The target population was cancer survivors who had completed primary treatment and had no active disease [17]. Neuropathic pain was defined as (1) a mean pain intensity the last week/month of ≥ 3 at a 11-point numerical rating scale (NRS), and (2) symptoms of neuropathy, or (3) neuropathic pain diagnosed by an experienced neurologist. The following study designs were included: guidelines, systematic reviews, and original studies (randomized controlled trials [RCT]), open or blinded, and cohort studies.

Since exercise interventions are shown to reduce neuropathic *symptoms*, an additional separate search on systematic reviews and meta-analyses only including RCTs addressing the effect of exercise interventions on reducing neuropathic *symptoms* was also conducted. In this search, cancer survivors were defined as individuals in active treatment or after cancer treatment.

### Sources of information

The searches were conducted in March 2024 (pharmacological and psychological treatment) and in April 2025 (exercise interventions) and all three searches were updated to include recent published literature in January 2026 through PubMed, PsycInfo, and EMBASE. Articles on cannabinoids were not included. Abstracts and grey literature were not included. Only articles in Danish and English were eligible. The search was restricted to studies published from 2004 to January 2026 to ensure the inclusion of evidence reflecting contemporary diagnostic practices and treatment approaches to neuropathic pain.

### Search

We used a general search query on cancer survivors and neuropathic pain as well as treatment-specific search terms for pharmacological, psychological and exercise interventions. A detailed search strategy can be found in the Appendix A1.

### Selection of sources of evidence

Our selection processes were visualized using PRISMA flow diagrams and Covidence review software was used to select sources of evidence. The screening and data extraction were performed by different reviewers according to treatment method. At least two reviewers independently performed the title and abstract screening and thereafter the full-text screening (pharmacological: ES, LV, AO, KS, psychological: CBM, PB, MJ, CJ, exercise interventions: BSR, SJ, LKN, IH). Conflicts were solved by a third person or by consensus between pairs.

### Quality appraisal

All included studies were assessed for methodological quality using a validated critical appraisal tool appropriate to each study design. In addition, study designs were categorized according to the Oxford Centre for Evidence-Based Medicine hierarchy of evidence to ensure a consistent classification across treatment modalities. The results of the quality evaluations can be found in the guideline (in Danish)

### Data charting process and data items

For each included publication, the following data items were registered: the first author, year of publication, study design, cancer type, sample size, ratio of patients who had completed treatment, study timeframe, etiology of cancer related neuropathic pain, interventions and whether the treatment had effect. Data extraction was performed by a single reviewer (ES) for the pharmacological data, two reviewers for the psychological data (CBM, PB) and four reviewers (BSR, SJ, LKN, IH) for data on exercise interventions.

### Synthesis of results

We included results from all eligible original studies and grouped the results according to treatment modality. When we identified a systematic review, we included eligible original studies that had been missing by our search by looking through the reference lists.

## Results

### Selection and characteristics of sources of evidence

**Figure 1:**
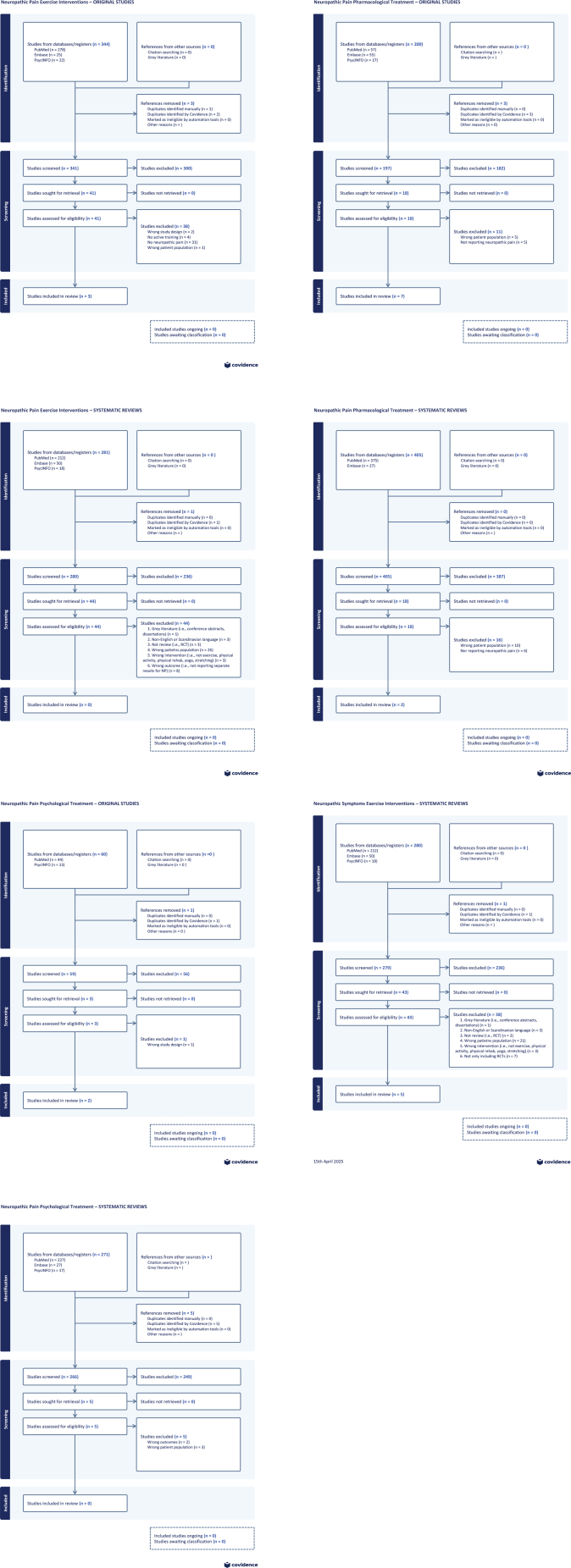
An overview of literature search, original and systematic reviews for exercise interventions, pharmacological interventions, psychological interventions, and for systematic reviews on exercise interventions for neuropathic *symptoms*.

A detailed description of the literature searches is presented in Figure 1 and Tables 1–3 present the study’s characteristics for the pharmacological, psychological, and exercise interventions, respectively. For the literature search on pharmacological treatments, 405 systematic reviews or guidelines and 200 original studies were identified. Of those, seven original studies were eligible, and only one systematic review and one guideline were relevant. Based on the reference list from the systematic review and the guideline, an additional four eligible studies were identified, adding to a total of 11 studies included. The literature search on psychological treatments identified 271 systematic reviews and 60 original studies. Here, two original studies and no systematic reviews were eligible. The literature search on exercise interventions revealed 281 systematic reviews and 344 original studies. Of those, no systematic reviews but three original studies were eligible. In total, 11 pharmacological (*n* = 1,209 patients), two psychological studies (*n* = 227 patients), and three exercise studies (*n* = 105 patients) were included.

**Table 1:** Original studies on pharmacological treatment of neuropathic pain.

Author, year	Design	Completed cancer treatment, (%)	Cancer type	Total number of patients included (I/C)	Follow-up time, weeks	Neuropathic pain etiology	Interventions	Effect, yes/no
Caraceni et al., 2004	RCT, blinded	100	Mixed, majority colorectal, lung and breast	80/41	1.5	Radiotherapy, surgery, tumor involvement	Gabapentin + concomitant morphine	Yes
Filipczak-Bryniarska et al., 2017	Cohort, open	100	Colorectal	18	12	CIPN	High-dose 8% capsaicin patch	Yes
Gewandter et al., 2014*^	RCT, blinded	100	Mixed, majority colorectal and breast	229/233	6	CIPN	Ketamine-amitriptyline cream	No
Hirayama et al., 2014*	RCT, crossover, open	100	Mixed, majority lymphoma and colorectal	34	4	CIPN	Duloxetine	Yes
Keskinbora et al., 2007 ^	RCT, open	100	NR	31/32	2	CIPN, surgery, tumor involvement	Gabapentin+ concomitant morphine	Yes
Velasco et al., 2021 ^	Cohort, open	100	Mixed, majority colorectal and breast	100	12	CIPN	Duloxetine	Yes (female, CIPN ≤ 6 months)
Vilholm et al., 2008^	RCT, crossover, blinded	100	Breast	27	4	Postmastectomy	Levetiracetam	No
Hincker et al., 2019	RCT, crossover, blinded	100	Mixed, majority colorectal and breast	26	4	CIPN	Pregabalin	No
Cheville et al., 2009	RCT, crossover, blinded	100	Mixed, majority lung and breast	28	4	Postoperative	5% lidocaine patches/placebo	No
Smith et al., 2013^	RCT, crossover, blinded	100	Mixed, majority gastrointestinal and breast	231	5	CIPN	Duloxetine	Yes
Singhal et al., 2025	RCT, open	100	Mixed, lung, oral cavity, breast, gall-bladder,	45/44	4	CIPN	Duloxetine or pregabalin	Yes

CIPN: chemotherapy induced peripheral neuropathy; I/C: intervention group/control group; NR: not reported; RCT: Randomized clinical trial.

* studies from guideline: Loprinzi CL, et al. Prevention and management of chemotherapy-induced peripheral neuropathy in survivors of adult cancers: ASCO guideline update. J Clin Oncol. 2020;38(28):3325–48.

^ studies from review: Guan J, Tanaka S, Kawakami K. Anticonvulsants or antidepressants in combination pharmacotherapy for treatment of neuropathic pain in cancer patients: a systematic review and meta-analysis. Clin J Pain. 2016;32(8):719–25.

**Table 2:** Psychological original studies on neuropathic pain.

Author, year	Design	Completed cancer treatment, (%)	Cancer type	Total number of patients included (I/C)	Follow-up time, weeks	Neuropathic pain etiology	Interventions	Effect, yes/no
Johannsen et al., 2016	RCT, open	100	Breast	67/62	24	CIPN, radiation, surgery	MBCT	Yes/no
Shergill et al., 2022	RCT, open	100	Breast	49/49	12	CIPN, surgery	MBCT	No

CIPN: chemotherapy induced peripheral neuropathy; MBCT: group-based, mindfulness-based cognitive behavioral therapy; RCT: Randomized clinical trial.

**Table 3:** Original studies, training interventions for treatment of neuropathic pain.

Author, year	Desig	Completed cancer treatment, (%)	Cancer type	Total number of patients included (I/C)	Follow-up time, weeks	Neuropathic pain etiology	Interventions	Effect, yes/no
Knoerl et al., 2024	RCT, open	100	Breast, gastrointestinal, gynecological	50/21	8	CIPN	Yoga	Yes
Wong et al., 2012	Cohort, open, pilot	100	Breast	8/8	24	Mixed	Cardiovascular exercise, upper body strength training	Yes
Lores-Peniche et al., 2024	Cohort, open, pilot	100	Breast	26	9	Mixed, 96% had neuropathic pain	Progressive resistance training and aerobic exercise modalities Pain neuroscience education program	Yes

CIPN: chemotherapy induced peripheral neuropathy; RCT: Randomized clinical trial.

### Results of individual sources of evidence

#### Pharmacological interventions for the treatment of cancer-related neuropathic pain in cancer survivors

Six studies were blinded RCTs, three were open RCTs, and two were cohort studies (Table 1). Seven studies included painful CIPN [18–21, 28, 33, 34], two with mixed etiologies (chemotherapy, radiation therapy, surgery, or tumor involvement) [29, 30] and two with postoperative neuropathic pain [31, 35]. Treatment included gabapentin [29, 30], duloxetine [19, 21, 28, 34], pregabalin [20, 28], levetiracetam [35], and topical treatment (capsaicin patches [18] or ketamine-amitriptyline cream [33]).

All open studies (at least in subgroups of patients) favored treatment (capsaicin patches, duloxetine or gabapentin + opioid vs. opioid only). For capsaicin patches, a reduction of pain intensity of > 83.9% ± standard deviation [SD]: 18.6 was observed after 12 weeks, *p* = 0.04 (*n* = 18) [18]. In the open cohort study by Velasco et al. (*n* = 100) investigating reduction in painful CIPN after 12 weeks of treatment with duloxetine, a change of 3 (range 1–7) in Patient Global Impression of Change (PGIC-score) was observed. This was not considered clinically meaningful (PGIC-score ≥ 5 was clinically relevant), and the dropout rate was large (37% due to side effects). However, in a multivariate analysis, female sex and short-lasting chemotherapy-induced neuropathic pain (≤ 6 months), the PGIC-score was ≥ 5 [19]. In the open randomized trial by Keskinbora et al., (*n* = 31, intervention, *n* = 32 control), gabapentin and an opioid treatment versus opioid treatment alone were investigated. Pain intensity for burning and shooting pain after 13 days was statistically significantly reduced in both groups, but the reduction in gabapentin + opioid group was larger compared to opioid only (for burning pain: −7.39 ± SD: 2.86 [gabapentin] vs. −5.78 ± SD: 2.35 [opioid only], *p =* 0.018; for shooting pain: −6.77 ± SD: 3.37 [gabapentin] vs. −4.66 ± SD: 2.80 [opioid only], *p* = 0.009) [29]. In an open, comparative RCT (duloxetine, *n* = 45 vs. pregabalin, *n* = 44), a statistically significant reduction in mean NRS were observed in both groups (from 7.04 ± SD: 0.903 to 4.04 ± 0.99 for duloxetine and 6.89 ± 0.920 to 4.91 ± 0.960 for pregabalin, both *p* < 0.001). Pregabalin had more adverse effects than duloxetine [28].

In the blinded RCTs, the following treatments versus placebo were investigated: pregabalin, gabapentin + concomitant morphine, 5% lidocaine patches, ketamine-amitriptyline cream, levetiracetam, and duloxetine (crossover) [20, 21, 30, 31, 33, 35]. Two RCTs found treatment superior to placebo, one investigating gabapentin + concomitant morphine versus placebo + concomitant morphine [30] and the other duloxetine versus placebo [21]. Caraceni et al. (*n* = 79 gabapentin, *n* = 41 placebo) found a difference in mean pain intensity after 10 days of treatment in favor of gabapentin. Adjusted mean pain score (0 (no pain) to 10 [worst pain]) was 4.6, standard error [SE]: 0.25 for gabapentin and 5.45, SE: 0.32 for placebo; *p* = 0.025 Analysis of Covariance (ANCOVA). There was no difference in baseline opioid use. Additional opioids were allowed, and patients receiving placebo used additional opioid doses more frequently than the gabapentin group (21.6% [gabapentin] vs. 35.8% [placebo], *p* = 0.056) [30]. In the randomized, double-blind, crossover study by Smith et al. (*n* = 231), treatment with duloxetine was favorable compared to placebo, measured as decrease in average pain intensity assessed by the Brief Pain Inventory-Short Form ‘average pain’ item (0 [no pain]–10 [as bad as one can imagine]). Decrease in average pain was 1.06 (95% confidence interval [CI]: 0.72–1.40) in the duloxetine group and 0.34 (95% CI: 0.01–0.66) in the placebo group (*p* = 0.003) after 5 weeks (i.e. after the initial treatment period) [21]. In the double-blind crossover study by Hincker et al., (*n* = 26) they found no significant difference between pregabalin and placebo after 28 days in reduction of average daily pain intensity (22.5% [pregabalin] vs. 10.7% [placebo], *p* = 0.23) or worst pain (29.2% [pregabalin] vs. 16.0% [placebo], *p* = 0.13) from baseline. However, in a post hoc analysis, a statistically significant larger reduction in worst pain in patients with oxaliplatin-induced CIPN (*n* = 518) treated with pregabalin compared to placebo (35.4% [pregabalin] vs. 14.6% [placebo], *p* = 0.04) was found [20]. Average weekly pain intensity did not differ between patients treated with lidocaine patches compared to placebo after 8 weeks in the double-blind, crossover RCT by Cheville et al., (*n* = 28) (NRS = 4.1 [lidocaine] versus 3.8 [placebo], *p* = 0.36) [31]. Similarly, no difference in pain intensity was found between levetiracetam and placebo in patients (*n* = 27) with post-mastectomy neuropathic pain (*p* = 0.83) [35].

#### Psychological interventions for the treatment of cancer-related neuropathic pain in cancer survivors

Two open RCTs were identified (Table 2) [36, 37]. Both studies evaluated the effect of group-based, mindfulness-based CBT versus a wait-list control group on neuropathic pain of mixed etiology (including chemotherapy or surgery-related neuropathic pain) in patients with breast cancer (*n* = 129 [36] and *n* = 98 [37]). Johannsen et al. found a statistically significant reduction in neuropathic pain based on the Short Form McGill Pain Questionnaire neuropathic subscale (Cohen’s *d* = 0.24, *p* = 0.036), although this effect was diluted after correction for multiple comparisons [36]. Shergill et al. found no effect of CBT on neuropathic pain symptoms evaluated by the Neuropathic Pain Symptom Inventory (*p* = 0.84) [37].

#### Exercise interventions for treatment of cancer-related neuropathic pain in cancer survivors

The evaluation was based on one open RCT and two cohort/pilot studies [38–40], Table 3. In the pilot study Lores-Peniche et al. (*n* = 26), investigated whether a pain neuroscience education program and a combined home and supervised exercise program with both progressive resistance training and aerobic exercise modalities (3 times a week, 9 weeks) could reduce neuropathic pain evaluated by the Douleur Neuropathic questionnaire. They found a statistically significant reduction from 5.96 ± SD: 1.83 at baseline to 2.31 ± 1.01 after 9 weeks, *p* = 0.001 [40]. The effect of a comprehensive health improvement program encompassing a 12-week cardiovascular exercise and upper body strength training program on neuropathic pain symptoms was investigated in the pilot study by Wong et al. (*n* = 8 intervention, *n* = 8 control). Here, a reduction in the sensory pain rating index from the McGill pain questionnaire was observed from 25% to 7% after 6 months compared to baseline (*p* < 0.05) (not reported for the control group). Compared to the control group, there was no difference in overall pain (both neuropathic and nociceptive pain), but an improvement in overall quality of life was observed (*p* < 0.01) [39]. In the open RCT from Knoerl et al. (*n* = 50 active, *n* = 21 control), yoga interventions (at least 12 yoga sessions over 8 weeks) were demonstrated to reduce worst CIPN pain intensity (NRS) compared to a control group (median change = −1.7, *p* < 0.001) [38].

#### Exercise interventions to improve neuropathic symptoms and balance in cancer survivors during or after treatment

We screened 280 studies, and of those, six systematic reviews and meta-analyses of RCTs were eligible, Table 4 [32–45]. All reviews focused on the efficacy of exercise for treatment of CIPN symptoms (*n* = 207 to 1,067). The patients suffered from different types of cancer (breast (majority), lung, and gastrointestinal cancer). All reviews included different exercise interventions, for example, balance, strength, sensory, yoga, stretching or aerobic exercises. The meta-analysis by Huang et al., (16 RCTs, *n* = 975) concluded that exercise can be a useful treatment in reducing neuropathic symptoms [41]. The studies had high heterogeneity and with some risk of publication bias [41]. Tamburin et al. (5 RCTs, *n* = 205) concluded that physical activity is effective in reducing neuropathy symptoms, although the RCTs had high heterogeneity, high risk of bias, and small sample sizes [43]. Also, Wang et al. (10 RCTs, *n* = 1,116) reported statistically significant effect of several exercise interventions in their network meta-analysis. They observed high heterogeneity for measures of CIPN symptoms but no significant publication bias based on Egger’s test [46]. On the contrary, Guo et al., did not find exercise interventions to be effective in reducing CIPN symptoms, but improvements in quality of life and physical symptoms, such as balance was reported (15 RCTs, *n* = 1,607). Heterogeneity and risk of bias were high [32]. Tanay et al. (13 RCTs, *n* = 743) also reported high heterogeneity and high risk of biases, but concluded that exercise intervention may be beneficial in reducing CIPN symptoms [44]. Similarly, Wang et al. (7 RCTs, *n* = 342) reported high heterogeneity and high risk of bias, and here the effect of exercise in reducing CIPN-symptoms was inconclusive [45].

**Table 4:** Systematic reviews and meta-analyses on exercise interventions for treatment of neuropathic *symptoms*. Cancer survivors for this search were defined as patients during or after cancer treatment.

Author, year	Design	During or completed treatment	Number of studies with training	Cancer type	Total number of patients included (I/C)	Etiology of neuropathy	Interventions, n studies	Effect, yes/no/inconclusive
Guo et al., 2023	Systematic review and meta-analysis	During and after	15	Mixed, e.g., lung, breast, gastrointenestinal, lymphoma	560/507	CIPN	Balance, 6 Strength, 6 Sensory, 4 Aerobic, 6	No
Huang et al., 2023	Systematic review and meta-analysis	During and after	16	Mixed, e.g., breast, lung, gastrointenestinal	539/436	CIPN	Balance, 3 Strength, 4 Sensory, 3 Aerobic, 3 Stretching, 1 Yoga, 1	Yes
Tamburin et al., 2022	Systematic review	During and after	5	Mixed, e.g., breast, colorectal, pancreatic, liver, bladder, prostate	106/99	CIPN	Balance, 4 Strength, 1 Sensory, 4 Aerobic, 1	Yes
Tanay et al., 2023	Systematic review	During and after	13	Mixed, e.g., breast, lymphoma, colorectal,	743 (both I and C)	CIPN	Balance, 7 Strength, 3 Sensory, 2 Aerobic, 3	Yes
Wang et al., 2022	Systematic review	During and after	7	Mixed, e.g., breast, colorectal, multiple myeloma	342 (both I and C)	CIPN	Balance, NR Strength, NR Sensory, NR Yoga, 3	Inconclusive
Wang et al., 2025	Systematic review and network meta-analysis	During and after	12	Mixed, e.g., breast, ovarian, colorectal, head and neck, lung	1,116	CIPN	Balance, 6 Strength, 14 Sensory, 8 Aerobic, 8 Yoga, 3	Yes

CIPN: chemotherapy induced peripheral neuropathy; I/C: intervention group/control group; NR: not reported; RCT: Randomized clinical trial.

## Discussion and conclusion

To our knowledge, this is the first review to provide an integrated overview of pharmacological, psychological, and exercise intervention strategies for neuropathic pain in cancer survivors who have completed primary treatment. Sixteen original studies published between 2004 and 2024 were identified (11 pharmacological, two psychological and three on exercise) addressing interventions to reduce neuropathic pain. Thus, our findings indicate a paucity of research focusing specifically on neuropathic pain treatment in this patient population. We also identified five systematic reviews (with or without meta-analyses) investigating exercise interventions to reduce neuropathic symptoms.

Based on the current literature we found that duloxetine is effective in reducing neuropathic pain in patients with painful CIPN and gabapentin + concomitant morphine reduces neuropathic pain in cancer survivors with neuropathic pain due to radiation therapy, surgery and tumor involvement. The two studies on mindfulness-based CBT showed no effect after correction for multiple comparisons. In addition, exercise interventions such as aerobic exercise and strength exercise are useful in both reducing neuropathic pain as well as neuropathic symptoms and can improve muscular strength and balance.

Many publications were excluded due to not having information on neuropathic pain or not exclusively including patients who had completed treatment for cancer. Across all treatment modalities, most of the patients had breast cancer and suffered from painful CIPN. This highlights the lack of knowledge in the field.

The recent (2020) interdisciplinary prevention and treatment guideline on CIPN, including painful CIPN by the American Society of Oncology (ASCO) concluded that duloxetine can be used for patients with painful CIPN who have completed chemotherapy, although the guideline [11] referred to studies where at least 50% of patients were in active chemotherapy treatment [34, 47, 48]. Furthermore, due to low quality of evidence, no recommendations on, for example, exercise therapy, gabapentin/pregabalin, and tricyclic antidepressants could be made. Regarding exercise therapy, based on preliminary results, Loprinzi et al. suggested that it may have an effect [11]. They did not mention psychological interventions.

Similarly, a systematic review on patients with breast cancer which only included studies on CIPN and with most patients in active treatment, demonstrated duloxetine as the only pharmacological treatment with substantial evidence to treat painful CIPN [49]. In addition, they conclude that exercise training may help to improve balance and neuropathy symptoms [49].

Since treatment of neuropathic pain is focused on symptom relief and not the triggering cause, it can be argued that the general NeuPSIG recommendation from 2025 of pharmacological treatment of neuropathic pain can also be applied among patients who have completed cancer treatment [6]. The NeuPSIG guideline is based on results from 314 different studies from different neuropathic pain etiologies, but no studies with cancer patients were included [6]. NeuPSIG recommend gabapentin, pregabalin, duloxetine, venlafaxine and amitriptyline as equal first-line treatments. Furthermore, opioids are only to be used in very special occasions and cannabinoids are not recommended [6]. This is in contradiction to the present literature search where only duloxetine for painful CIPN and to some degree gabapentin for mixed etiology neuropathic pain had effect (in blinded RCTs). However, the other first-line drugs have not been tested in blinded RCTs in cancer survivors who have completed treatment. In the ASCO guideline cannabinoids are not recommended and they did not mention opioids [6, 11].

Unfortunately, only two studies on psychological treatment were eligible and were with conflicting results. In mindfulness-based CBT patients practice to relate to bodily sensations and emotional discomfort with an attitude of acceptance, openness and non-judgment [50]. The rationale is that targeting cognitive and affective dimensions of the pain experience with psychological interventions may facilitate the ability to cope more adaptively. More studies are needed to conclude whether CBT is effective in relieving cancer-related neuropathic pain and an online self-help intervention based on Acceptance and Commitment Therapy (ACT) is under development with the aim to reduce pain interference in cancer survivors experiencing painful chronic CIPN [51]. Looking at the broader literature regarding pain in cancer patients and survivors, there are numerous studies showing positive effects of a variety of psychosocial interventions [26, 27]. Although there is a sparsity of studies investigating the efficacy of specifically psychological interventions. From a psychological perspective, the aim is to target the way an individual is relating to the pain and to redefine a meaningful life despite pain, rather than altering the pain per se. This suggests that the type of pain may bear less clinical importance in psychological pain management.

The eligible studies on exercise interventions did all present a positive impact on neuropathic pain, suggesting an effect of training, although more detailed studies and with higher number of participants are needed.

For relieving neuropathic symptoms and improving balance (i.e. not neuropathic pain, but neuropathic symptoms in general), exercise interventions proved effective. We only found reviews including patients with CIPN, thus limiting the generalizability. It was not possible to find systematic reviews that only included patients who had completed treatment. Also, due to minimal side effects and preliminary promising results, training interventions are recommended in relieving neuropathic symptoms.

### Strengths and limitations

The strength of this scoping review is the multidisciplinary approach thus making a comprehensive coverage on literature on treatment of neuropathic pain in cancer survivors. A limitation is that the study was not pre-registered at PROSPERO, limiting transparency.

## Conclusion

This scoping review provides the current state of knowledge on the interdisciplinary treatment of neuropathic pain among cancer survivors who have completed oncological treatment. The literature of this topic is sparse, and thus our study underlines the need for more research in this field. For pharmacological treatment, we not only recommend to use the recently updated NeuPSIG recommendations for neuropathic pain but to also consider the use of duloxetine as first-line treatment for painful CIPN. In addition, the primary indication as well as the side effects in choice of prescription should be also considered. It was not possible to make any conclusion regarding the effect of psychological interventions on neuropathic pain due to a low number of studies. Exercise interventions show promising effects on neuropathic pain and neuropathic symptoms and are recommended. Thus, an interdisciplinary approach to treating neuropathic pain in cancer survivors is highly relevant, but further studies are needed.

## Supplementary Material



## Data Availability

The datasets generated during and/or analyzed during this study are available from the first author upon reasonable request.
