# Supplementary material for: Treatment of neuropathic pain in cancer survivors: a scoping review of pharmacological, exercise, and psychosocial interventions
Source: Acta Oncol. 2026 Apr 24;65:45347. doi: 10.2340/ao.v65.45347 (PMC13127108; doi:10.2340/ao.v65.45347)
Supplement: Supplementary file 1 [file AO-65-45347-s1.pdf]

Supplementary material has been published as submitted. It has not been copyedited, or typeset by Acta Oncologica

Appendix

Treatment of Neuropathic Pain in Cancer Survivors: A Scoping Review of Pharmacological, Exercise, and Psychosocial interventions

Ellen Lund Schaldemose et al.

A1: Literature search:

The specific query used was:[Oncolog* Patient*] OR [Cancer Surviv*] OR [Cancer Patient*] OR [malignan*] OR [Neoplasm] OR [Cancer*] OR [carcino*] AND [Treatment related sequelae] OR [Neuropathic pain] OR [chemotherapy-induced peripheral neuropathy] OR [chemotherapy-induced neuropathic pain] OR [neuropathic pain after cancer surgery/treatment] as well as the following treatment specific search terms:

Pharmacological treatment: Pharmacologic actions OR Analgesia OR Pharmacotherapy OR Antidepressive Agents OR Tricyclic OR Serotonin and Noradrenaline Reuptake Inhibitors OR Pregabalin OR Gabapentin OR Opioid OR Cannabis OR Lamotrigine OR Lidocaine OR Topical OR Capsaicin.

Psychological treatment: "Cognitive behavioral therapy" OR CBT OR "acceptance and commitment therapy" OR ACT OR "mindfulness-based intervention" OR "mindfulness-based therapy" OR MBSR OR MBCT OR psychoeducation OR "psycho-education" OR psychotherapy OR psychoscocial OR psychological OR counseling

Exercise interventions: [exercise] OR [physical activity] OR [acute exercise] OR [exercise training] OR [aerobic exercise] OR [resistance training] OR [strength training] OR [running] OR [yoga] OR [balance training.
